# Direct nanopore RNA sequencing of umbra-like virus-infected plants reveals long non-coding RNAs, specific cleavage sites, D-RNAs, foldback RNAs, and temporal- and tissue-specific profiles

**DOI:** 10.1093/nargab/lqae104

**Published:** 2024-08-16

**Authors:** Philip Z Johnson, Jason M Needham, Natalie K Lim, Anne E Simon

**Affiliations:** Department of Cell Biology and Molecular Genetics, University of Maryland, College Park, USA; Department of Cell Biology and Molecular Genetics, University of Maryland, College Park, USA; Department of Cell Biology and Molecular Genetics, University of Maryland, College Park, USA; Department of Cell Biology and Molecular Genetics, University of Maryland, College Park, USA

## Abstract

The traditional view of plus (+)-strand RNA virus transcriptomes is that infected cells contain a limited variety of viral RNAs, such as full-length (+)-strand genomic RNA(s), (–)-strand replication intermediate(s), 3′ co-terminal subgenomic RNA(s), and viral recombinant defective (D)-RNAs. To ascertain the full complement of viral RNAs associated with the simplest plant viruses, long-read direct RNA nanopore sequencing was used to perform transcriptomic analyses of two related umbra-like viruses: citrus yellow vein-associated virus (CY1) from citrus and CY2 from hemp. Analysis of different timepoints/tissues in CY1- and CY2-infected *Nicotiana benthamiana* plants and CY2-infected hemp revealed: (i) three 5′ co-terminal RNAs of 281 nt, 442 nt and 671 nt, each generated by a different mechanism; (ii) D-RNA populations containing the 671 fragment at their 5′ends; (iii) many full-length genomic RNAs and D-RNAs with identical 3′end 61 nt truncations; (iv) virtually all (–)-strand reads missing 3 nt at their 3′ termini; (v) (±) foldback RNAs comprising about one-third of all (–)-strand reads and (vi) a higher proportion of full-length gRNAs in roots than in leaves, suggesting that roots may be functioning as a gRNA reservoir. These findings suggest that viral transcriptomes are much more complex than previously thought.

## Introduction

The transcriptomes of plus (+)-strand RNA viruses are thought to be composed of a limited variety of (+)-strand and (–)-strand transcripts. These include protein-coding transcripts such as the (+)-sense genomic (g)RNA(s), which contain the entirety of the viral genome and can typically initiate an infection independent of other viral transcripts, and can include 3′ co-terminal subgenomic (sg)RNAs for translation of downstream ORFs (1,2). Complementary (–)-sense gRNA and sgRNA transcripts are also present, along with recombinant defective (D)-RNAs usually generated by the viral-encoded RNA-dependent RNA polymerase (RdRp) and composed of (at least) 5′ and 3′ co-terminal (+)-sense gRNA segments (3,4). Junctions between 5′ and 3′ segments of D-RNAs frequently share overlapping sequences that are repeated in the viral gRNA but occur only once in the D-RNA itself. The presence of this shared sequence is compatible with a template-switching mechanism for D-RNA generation, whereby the viral RdRp upon transcribing the 5′ D-RNA segment ‘jumps’ to continue (+)-strand synthesis at the start of the 3′ D-RNA segment (3,4). D-RNAs are generally non-coding, may accumulate to higher levels than the parental gRNA over time (5,6), and can reduce or enhance the virulence of the parental virus (7,8).

In addition to the expected transcriptome constituents, there are sporadic reports of less well characterized viral transcripts. For example, (+)-strand gRNAs can be present with 3′ terminal deletions (∼100 nt or less) that no longer contain RNA structural elements critical for replication and/or translation (9). Diverse nonreplicative recombination events (i.e. not involving the viral RdRp) between viral genome segments and/or host RNAs have also been reported among picornaviruses and flaviviruses. Although it is not yet known how these events occur, they may contribute to viral evolution (10). Furthermore, virus-derived long non-coding (lnc)RNAs (loosely defined as any gRNA fragment ≥200 nt without an apparent coding capacity) can function as inhibitors of host defense/immune responses (11,12). For example, citrus tristeza virus (CTV) generates a highly abundant 5′ co-terminal lncRNA early in the infection that is synthesized by the viral-encoded RdRp and functions to inhibit salicylic acid synthesis, thus blocking a key component of the plant antiviral defense response (13,14). Similarly, a 3′ co-terminal lncRNA produced by beet necrotic yellow vein virus A inhibits antiviral RNA silencing to promote systemic spread (15), and animal flaviviruses produce a 3′ co-terminal lncRNA that functions to inhibit RNA silencing and reduce the effects of type I interferon (16–18). Barley yellow dwarf virus (BYDV) expresses a 3′ co-terminal lncRNA that attenuates host protein translation by sequestering translation initiation factor eIF4G (12).

Other components of viral transcriptomes are plus/minus (±)-foldback RNAs (also known as copyback or snapback RNAs) that are typically composed of 5′ co-terminal (+)-strand sequence joined to the (–)-strand complement (4,19). (±)-foldbacks are thought to arise when the viral RdRp only dissociates from the template after transcription terminates and re-initiates synthesis using the nascent (+)-strand as the template thus generating almost fully double-stranded RNAs (4). The foldback RNAs generated by (–)-strand RNA viruses can accumulate to high levels (5,6) and are associated with persistent viral infection as primary stimulants of the host immune response (4,20–23). Foldbacks generated by (+)-strand RNA viruses are typically found at much lower concentrations and their roles in viral infection, if any, remain unknown (19,24,25).

Direct RNA nanopore sequencing (DRS) generates nearly full-length reads of individual transcripts (26,27) thus enabling determination of the full transcriptomic complexity in an RNA sample. During DRS, RNA strands are threaded through membrane-bound nanopore proteins with the resulting fluctuations in the electrical signal allowing for identification of nucleotides. DRS sequences RNA in the 3′-to-5′ direction with reads usually terminating ∼13 nt short of the 5′end, and results in an average per-nucleotide error rate of ∼9–12% (26). Since individual RNA strands are directly sequenced, DRS is uniquely suited for studying molecules that are subgenomic in length or products of recombination, and can also identify discrete viral transcripts present at low levels that would otherwise be missed using a standard RNAseq approach (26,27).

**Figure 1.**
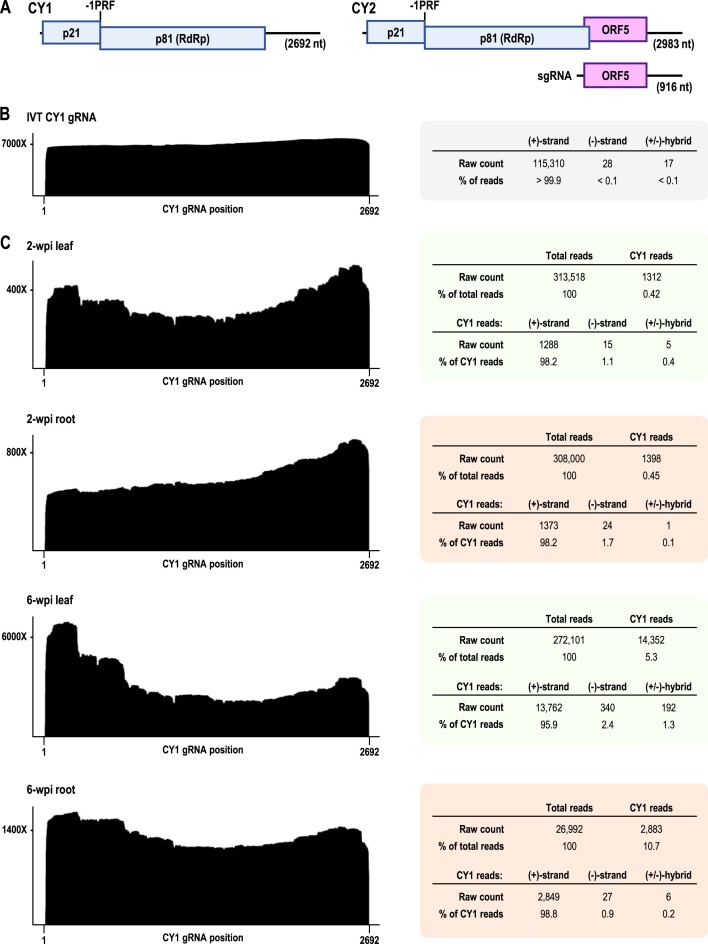
Coverage maps and read counts for *in vitro* transcribed (IVT) CY1 gRNA and the CY1 (+)-strand transcriptome in infected *N. benthamiana* leaves and roots. (**A**) Gene organization of CY1 and CY2 gRNAs and CY2 sgRNA. Sites of -1 programmed ribosomal frameshifting (-1PRF) are marked. (**B**) DRS analysis of CY1 gRNA IVT. Total CY1 read counts are shown to the right. Most IVT reads were missing the 5′ terminal ∼13 nt, which is typical for DRS reads, and the average per-nucleotide accuracy was 90.7%, which is comparable to values obtained in recent reports (26,42,43). (**C**) DRS analyses of CY1 in total RNA samples from 2- and 6-wpi leaves and roots. The height of these (+)-strand RNA coverage maps at each position along the CY1 genome indicates the number of reads that contain (i.e., ‘cover’) that position. For example, a coverage height of 7000× at position 100 indicates that 7000 reads contain position 100 of the CY1 genome.

For an initial assessment of plant (+)-strand RNA virus transcriptomes, we chose 2692 nt citrus yellow vein-associated virus (CY1; previously known as CYVaV) from citrus, and related (90% sequence similarity) CY2 (2983 nt) from *Cannabis sativa* (hemp) because of their limited size and coding capacity. CY1 and CY2 are known as umbra-like viruses (ULVs), a recently discovered grouping of (+)-strand RNA viruses that encode an umbravirus (family *Tombusviridae*)-related RdRp and have umbravirus-like 3′ terminal RNA structures (28–30). As with umbraviruses, all ULVs encode a replicase-associated protein (ORF1) that can be extended by -1 programmed ribosomal frameshifting to generate the RdRp (ORF2) (Figure 1A). Some ULVs only encode these two replication-associated proteins, while others contain one or two additional ORFs of different origins that are either known or predicted to be translated from sgRNAs (30). Whereas umbraviruses are only found in plants in the presence of a helper virus due to lack of an encoded capsid protein, most ULVs (including CY1 and CY2) are not associated with a discernible helper virus (30) and full-length transcripts are capable of establishing independent systemic infections (31,32). Recent studies of high-throughput sequencing data from field samples of diverse plant species suggest that ULVs are abundant in nature, but have only recently been discovered because most are present in plants without discernible symptoms (30,33–41).

ULVs are currently divided into two groups, with Group 2 containing three classes (30). Group 2/Class 1 ULVs contain only ORFs 1 and 2, whereas nearly all Class 2 ULVs including CY2 contain an additional ORF known as ORF5 (to distinguish it from umbravirus ORFs 3 and 4 that encode movement-related proteins). ORF5, which partly overlaps with ORF2, is expressed from an sgRNA and encodes a capsid-like protein. Thus ULVs are the first recognized plant viruses without encoded movement proteins that use host RNA movement proteins to independently infect plants (31). CY1 is an unusual member of Class 2 as it no longer contains ORF5 due to deletions and other alterations that also eliminate sgRNA synthesis (Figure 1A), yet is still fully capable of systemically infecting plants (29).

Here, we employed DRS to map the transcriptomes of CY1 and CY2 in infected *N. benthamiana* leaves and roots at multiple timepoints, and CY2 in an infected field sample. In addition to (+)- and (–)-sense full-length gRNAs (and sgRNAs for CY2), we identified three novel 5′ co-terminal RNAs of 281, 442 and 671 nt. D-RNAs were also found, with the majority containing the 671 nt fragment (F671) as the 5′ portion. Many different foldback RNAs were identified, with the (+)-sense portion frequently terminating at position 671, suggesting that F671 is RdRp-derived. A prominent cleavage site that was likely enzymatically generated was associated with the 442 nt fragment (F442) but not with the 281 nt fragment (F281), suggesting that different mechanisms generated these smaller abundant transcripts. We also found a much higher proportion of full-length gRNA in roots rather than in leaves, suggesting that roots are a possible reservoir for viral gRNA. The CY2 transcriptome in naturally infected hemp contained high levels of a single D-RNA in contrast with the three D-RNAs found in CY2-infected *N. benthamiana*, reflecting additional D-RNA selection that occurred during long-term plant infection. Additionally, (–)-sense reads, whether alone or as portions of foldbacks, were missing their 3′ terminal three nucleotides, suggesting that the complementary (+)-strand residues are synthesized by a non-templated mechanism. These results suggest that the transcriptomes of even the simplest (+)-strand RNA plant viruses are more complex than previously thought.

## Materials and methods

### In vitro transcription of CY1 RNA

pET17b (44) containing full-length CY1 gRNA sequence (GenBank: JX101610) immediately downstream of a T7 promoter was linearized with HindIII (New England Biolabs) and used as template for *in vitro* transcription using T7 polymerase. In vitro transcribed CY1 gRNA sample volume was raised to 100 μl with ddH_2_O followed by addition of 100 μl of 5M LiCl and incubation at –20°C for 30 min. Samples were centrifuged at top speed for 30 min at 4°C followed by a 75% ethanol wash, air drying, and resuspension in ddH_2_O. IVT templates for smaller RNAs were PCR amplified from plasmid containing either full-length CY1 gRNA sequence or D-RNA sequence using OneTaq DNA polymerase (New England Biolabs) in standard buffer and a forward primer containing a 5′ co-terminal T7 RNA polymerase promoter. PCR products were ethanol precipitated and then used as template for *in vitro* transcription. F1600 and D-RNA were LiCl precipitated, while F281 was ethanol precipitated due to its smaller size resulting in less efficient LiCl precipitation. F671 RNA was LiCl precipitated when used as a northern blot size marker and ethanol precipitated when used in the DRS sequencing experiment.

### In-line probing of IVT CY1 gRNA

Three micrograms of IVT CY1 gRNA was incubated at 25°C for 90 min in RNA folding buffer (80 mM Tris–HCl [pH 8.0], 11 mM Mg(CH_3_COO)_2_, 160 mM NH_4_Cl). RNA was then purified using RNAClean XP beads following manufacturer's instructions and visualized on an ethidium-stained 1% agarose gel to measure the approximate level of in-line autocleavage (about 75%). The in-line autocleaved RNA sample was then subjected to DRS sequencing beginning with the poly(A) tailing step.

Following DRS sequencing, 5′ terminal positions of reads were counted. Owing to the tendency of DRS to terminate prematurely (∼13 nt away from the 5′end) (26), counts of 5′ terminal read positions were shifted 13 nt upstream. To account for signal decay going 3′-to-5′ across the length of the viral genome (since DRS sequences in the 3′-to-5′ direction, and is unable to sequence the 5′ fragments initially produced by autocleavage of RNA due to lack of a 3′ terminal hydroxyl group), a logarithmic signal decay correction was applied. To dampen the effects of variation in precisely where DRS sequencing terminates (relative to the 5′end of an RNA molecule), counts of read 5′ terminal positions were averaged using a sliding window approach with a window size of 5 nt.

### Agroinfiltration of *N. benthamiana* plants with CY1 and CY2

*Agrobacterium tumefaciens* strain GV3101 was transformed by electroporation with binary vector pCB301 containing full-length CY1 (JX101610) or CY2 gRNA sequence (31) immediately downstream of duplicated cauliflower mosaic virus 35S promoters and immediately upstream of a hammerhead ribozyme sequence. Transformed *A. tumefaciens* cultures were grown to an OD between 1.0 and 1.2 in 0.5 l of Luria-Bertani broth supplemented with antibiotics [rifampicin (20 μg/ml) and kanamycin (50 μg/ml)] over the course of ∼18 h, along with *A. tumefaciens* cultures transformed with a standard RNA silencing suppressor [p19 (45) or p14 (46)]. *A. tumefaciens* cultures were centrifuged at 5K rpm for 10 min using a Sorvall SLA-1500 rotor, resuspended in infiltration buffer (10 mM MgCl_2_; 10 mM MES; 100 ng/ml acetosyringone) at an OD of 1.2 for viral cultures and 0.4 for RNA silencing suppressor cultures, mixed in a 1:1 ratio of viral culture to RNA silencing suppressor culture, and incubated for 2 h at room temperature. *N. benthamiana* plants containing six true leaves were then submerged inverted in the mixed *A. tumefaciens* cultures and vacuum infiltrated using a negative pressure of –25 Hg for 30 s. Plants were grown at 25°C and with a 12 h light cycle. Systemic leaf and primary root stalk samples were harvested from infiltrated plants at 2-, 6- and 14-weeks post infiltration (wpi).

### Extraction of RNA from infected plant samples

Total RNA was extracted from infected plant samples using TRIzol reagent (Invitrogen) following the manufacturer's instructions. Root samples were thoroughly ground with a mortar and pestle after being frozen in liquid nitrogen immediately prior to TRIzol extraction. Following TRIzol extraction, extracted root RNA samples were LiCl precipitated twice to remove excess polysaccharides in the RNA samples. All extracted RNA samples were purified using RNAClean XP beads (Beckman Coulter) and analyzed by ethidium bromide-stained agarose gel electrophoresis prior to any downstream procedures.

### Poly(A) tailing of RNA

Approximately 500 ng of RNA was mixed with ddH_2_O to a volume of 15.5 μl. Two microliters of 10X buffer, 2 μl of 10 mM ATP, and 0.5 μl of *Escherichia coli* poly(A) polymerase (New England Biolabs, enzyme and buffer) were then added. Reactions were incubated at 37°C for 3–5 min and then terminated by addition of 5 μl of 50 mM EDTA. Poly(A) tailed RNA was purified using RNAClean XP beads (Beckman Coulter) following manufacturer's instructions and resuspended in 12–16 μl of ddH_2_O.

### Direct RNA nanopore sequencing

For all sequencing runs, sequencing libraries were prepared from poly(A)-tailed RNA samples using the direct RNA sequencing kit (SQK-RNA002) following manufacturer's instructions and including the reverse transcription step to generate RNA:cDNA hybrids. Sequencing runs (6–18 h) were performed using version R9.4.1 flow cells and a MinION Mk1B device. Used flow cells were cleaned between runs using the flow cell wash kit (EXP-WSH004) following the manufacturer's instructions.

### Basecalling of DRS reads and alignment to the CY1 and CY2 reference genomes

Since DRS sequencing runs for this report were performed over a 2-year period, ranges of software versions that were used are given. MinKNOW (v22.03.6 to v23.11.25) was used for basecalling of nanopore sequencing reads using the standard quality score threshold of 7 for direct RNA sequencing (corresponding to at least 80% read accuracy) and using Guppy (v6.1.7 to v6.5.7) or Dorado (v7.0.8 to v7.2.13) basecallers. All DRS reads were basecalled using the high-accuracy ‘2020–09-07_rna_r9.4.1_minion_256_8f8fc47b’ model, with the one exception of DRS reads for the IVT RNA size markers experiment (Figure 8B and C), which were basecalled using the high-accuracy ‘rna002_70bps_hac@v3’ model. Reads meeting the quality score threshold were aligned to CY1 and CY2 gRNA sequences using minimap2 (v2.24-r1122 to v2.28-r1209) running within the EPI2ME Labs software (v22.06.01 to v23.05–01). Reads with primary alignments to viral gRNA sequences were deemed to be viral in origin and extracted using samtools (v1.15.1 to v1.20). Viral reads were then realigned to viral gRNA sequences using the BLAST+ command-line tool (v2.12.0 to v2.14.1) (47). BLAST+ software was set to format its output in JSON, which was then analyzed as described below.

### Analysis and visualization of DRS sequencing data

BLAST JSON output for all DRS sequencing runs were analyzed using custom Python (v3.11.4) scripts created for this report and all visualizations were created using the Matplotlib library (v3.7.2) (48). All custom scripts have been deposited in the following GitHub repository: https://github.com/pzhaojohnson/nar-gab.johnson-etal. Note that all scripts require their corresponding BLAST alignment JSON output(s) and/or sequencing data that they reference to be present in the same directory as the script in order to run. (All BLAST alignment JSON outputs and sequencing data are provided as supplementary materials.) In general, BLAST JSON output was parsed using the built-in Python json library.

### Coverage maps and skew quantitation

Coverage maps were generated using the Matplotlib 'hist' function (e.g. by plotting a histogram of the unique viral genome positions covered by each read for a given sequencing dataset). Quantitation of coverage map 5′ and 3′ skews was done using the following formulas:

5′ skew = (maximum 5′ half coverage) / (median viral genome position coverage) – 1

3′ skew = (maximum 3′ half coverage) / (median viral genome position coverage) – 1

For example, for 6-wpi CY1-infected leaf, the median viral genome position is 1346 and its (+)-strand coverage was 2501 and the maximum 5′ half (+)-strand coverage was 6912, resulting in a 5′ skew of 1.76 (i.e. 176%). To give another example, if coverage of the median viral genome position were equal to the maximum coverage of the 5′ half of the viral genome, then 5′ skew would be 0%. Also, since the genome length of CY2 is 2983 nt and odd, its median viral genome position coverage is calculated as the average coverage of positions 1491 and 1492.

### Read alignment plots

**Figure 2.**
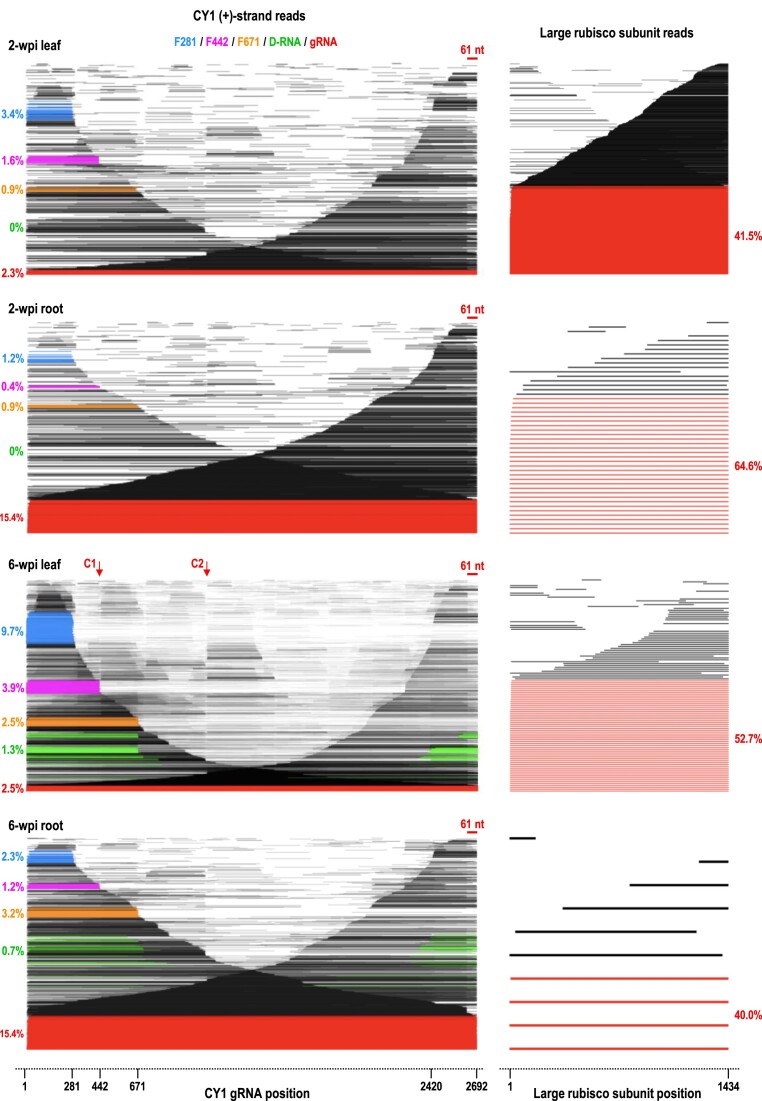
Read alignment plots for (+)-strand CY1 and large subunit ribulose bis-phosphate carboxylase mRNA in 2- and 6-wpi leaves and roots. Left, read alignment plots of all CY1 (+)-strand reads at different times and in different tissues. Reads are ordered vertically according to their alignment length with each read assigned a unique Y axis position and positioned on the X axis based on their alignment to the viral genome. CY1 reads are color coded as follows: full-length gRNA (red), D-RNA (green), F281 (blue), F442 (magenta), F671 (orange). Percentages of reads corresponding to each of these constituents are given. No D-RNA reads were detected at 2-wpi. Throughout this report, reads are considered to correspond to single segment RNAs (i.e. full-length gRNA, F281, F442, F671 and rubisco mRNA) if they possessed only a single segment with the 5′end of their alignment within 30 nt of position 1 of the viral gRNA/rubisco sequence, and the 3′end of their alignment within 10 nt of the RNA′s 3′end (DRS sequences 5′ terminal residues with less precision than 3′ terminal residues). Reads were considered to correspond to a D-RNA if they possessed two segments with the 5′ segment beginning within 30 nt of position 1 and the 3′ segment ending within 10 nt of the 3′ end, with an overall covered length of less than 2000 nt. Right, alignment plots of host large subunit rubisco mRNA reads in the same samples as a control for RNA integrity. Full length rubisco reads are colored red. Note that with the exception of 2-wpi roots, many CY1 reads that cover 3′ terminal sequences are missing the 3′ terminal 61 nt (see Supplementary Figure S2A for an enlargement). Prominent cleavage points (C1 and C2) are labeled for the 6-wpi leaf reads.

Plots of reads and their alignments to the viral genome (e.g. in Figure 2) were created using the Matplotlib ‘plot’ function. Reads were classified as single alignment categories (e.g. gRNA, F281, F442, F671) if they: (i) had a 5′ terminal covered position within 30 nt of the 5′end of the target RNA boundary; (ii) had a 3′ terminal covered position within 10 nt of the 3′end of the target RNA boundary since DRS sequences 3′ends more precisely than 5′ends; and (iii) the read aligned to a single, continuous segment of the reference genome. Reads were considered D-RNAs if they: (i) aligned to two, discontinuous segments of the reference genome; (ii) had a 5′ terminal covered position within 30 nt of position 1 of the viral genome; (iii) had a 3′ terminal covered position within 10 nt of the 3′end of the viral genome and (iv) aligned to <2000 nt of the viral genome. Reads corresponding to identified transcripts were colored (e.g. red for full-length gRNA, blue for F281, green for D-RNA) while reads corresponding to unclassified transcripts were colored black and assigned a transparency inversely related to the relative abundance of the read.

### RNA folding and measuring hairpin-ness

Folding of read sequences was performed using RNAfold (Vienna RNA software suite v2.6.4) run locally using default folding parameters (49). Predicted RNA hairpin structures were measured as the mountain plot, where the height of a structure was divided by the total length of the structure's sequence. Thus, a fully complete hairpin (i.e. one with zero unpaired bases in its loop) would have a score of 50%.

### Northern blotting of extracted RNA samples

As previously described (50), extracted RNA was subjected to electrophoresis on an ethidium bromide-stained, non-denaturing 1.5% agarose gel. RNA was then transferred to a positively charged nylon membrane by capillary action overnight and then cross-linked to the nylon membrane by exposure to UV light. Radiolabeled probes consisted of single-stranded DNA oligonucleotides labeled at their 5′ ends using T4 polynucleotide kinase (New England Biolabs) and $\gamma - {\mathrm{P}}32 -$ATP (Revvity). Hybridizations were performed overnight at 50°C. Hybridized membranes were imaged using an Amersham Typhoon Biomolecular Imager.

### Two-dimensional RNA structure drawing

All two-dimensional RNA structure drawings were generated using the RNAcanvas web app (51).

## Results and discussion

### DRS of *in vitro* transcribed CY1 gRNA

Before using DRS to sequence CY1 and CY2 transcriptomes *in planta*, we first assessed the ability of DRS to sequence full-length CY1 transcripts that were synthesized *in vitro* (IVT). Basecalling for DRS runs was performed using Guppy (high-accuracy model) using the standard quality cutoff score of 7. CY1 reads were extracted using minimap2 (52) and aligned to the CY1 reference genome using locally run BLAST+ (47). Nearly all reads obtained from IVT sequencing were of the expected (+)-strand, with less than 0.1% of the reads containing (–)-strands or both (+)- and (–)-strand segments (Figure 1B, right). T7 RNA polymerase can infrequently use RNA as a template (53) and can also generate low levels of foldback transcripts (54,55), accounting for the (–)-strand and hybrid reads.

The IVT coverage map was nearly uniform with a 3′ skew of 13% (see Materials and Methods for coverage map skew quantification method) (Figure 1B, left). While this coverage map would suggest that the majority of reads were full-length, a read alignment plot, which displays the alignments of individual reads ordered by alignment length, revealed that only 42% of reads were full-length despite full-length transcripts being the major transcript on the agarose gel (Supplementary Figure S1B and C). The subgenomic-length reads likely resulted from early T7 polymerase termination, premature termination of DRS sequencing, and/or were products of random cleavages that took place during sample preparation. Most subgenomic-length reads were either 5′ or 3′ co-terminal and thus almost perfectly ‘cancelled out’ in the coverage map, resulting in near uniformity of coverage. This preliminary finding demonstrates that coverage maps, which are routinely used to display DRS sequencing outputs, do not provide reliable information about the lengths of individual transcripts, which are more accurately represented by read alignment plots.

### DRS of CY1-infected *N. benthamiana* leaves and roots

*Nicotiana benthamiana* plants possessing six true leaves were vacuum infiltrated with *A. tumefaciens* T-DNA containing full-length CY1 cDNA downstream from duplicated cauliflower mosaic virus 35S promoters. Young systemic leaves and primary root stalks were harvested at 2- and 6-weeks wpi, and DRS was performed on total extracted RNA (Figure 1C). At 2-wpi, CY1 reads comprised 0.42% of the total leaf reads and 0.45% of the total root reads. Among CY1 reads at 2-wpi, (+)-strand reads comprised 98.2% for both leaf and root samples, (–)-strand reads were somewhat lower for leaves (1.1% versus 1.7% for roots), and (±)-hybrid reads were higher for leaves (0.4% versus 0.1%). At 6-wpi, CY1 levels increased to 5.3% of total leaf reads and 10.7% of total root reads. 6-wpi leaves contained reads that were 95.9% (+)-strands, 2.4% (–)-strands, and 1.3% (±)-hybrids. In contrast, 6-wpi roots contained a higher percentage of (+)-strand reads (98.8%), with fewer (–)-strand reads (0.9%) and (±)-hybrid reads (0.2%). The (+)-strand coverage maps for 2- and 6-wpi leaves and roots differed from that of CY1 gRNA IVT by overall shape, degrees of 5′/3′ skew, the presence of major drop-offs, and small dips in coverage known as grooves (Figure 1C and Supplementary Figure S2).

### Major (+)-strand transcripts

Read alignment plots of individual CY1 (+)-strand reads at 2- and 6-wpi revealed surprisingly low levels of full-length gRNA in leaves (∼2.5% of CY1 reads; Figure 2, left in red). Full-length CY1 read percentages from roots were over 6-fold higher (15.4% of CY1 reads) at both timepoints. To determine if low levels of full-length reads reflected significant fragmentation during sample preparation, read alignment plots were generated for ribulose bis-phosphate carboxylase (rubisco) large subunit mRNA reads present in the sequencing data from the same run (Figure 2, right). Rubisco reads exhibited a 3′ skew, with percentages of full-length reads ranging from 40% to 65%. This suggests that the low levels of CY1 full-length gRNA reads were not a consequence of significant experimental fragmentation prior to DRS.

Three major 5′ co-terminal subgenomic-length RNAs were present in 2-wpi leaves that increased in prevalence at 6-wpi (Figure 2). These 5′ co-terminal fragments terminated at or near positions 281 (F281; blue), 442 (F442; magenta), and 671 (F671; orange). F281 comprised 9.7% of the leaf CY1 reads at 6-wpi, accounting for the significant early drop-off (labeled DO2) in the coverage map (Supplementary Figure S2A, top). Nearly 4% of the CY1 reads in 6-wpi leaves were F442, corresponding to sharp groove Gr2 in the coverage map, and F671 was 2.5% of the CY1 reads, corresponding in part to DO3. Since F281, F442 and F671 lack 3′UTR elements required for efficient translation of CY1 gRNA (44), these 5′ co-terminal fragments likely represent virus-derived lncRNAs.

**Figure 3.**
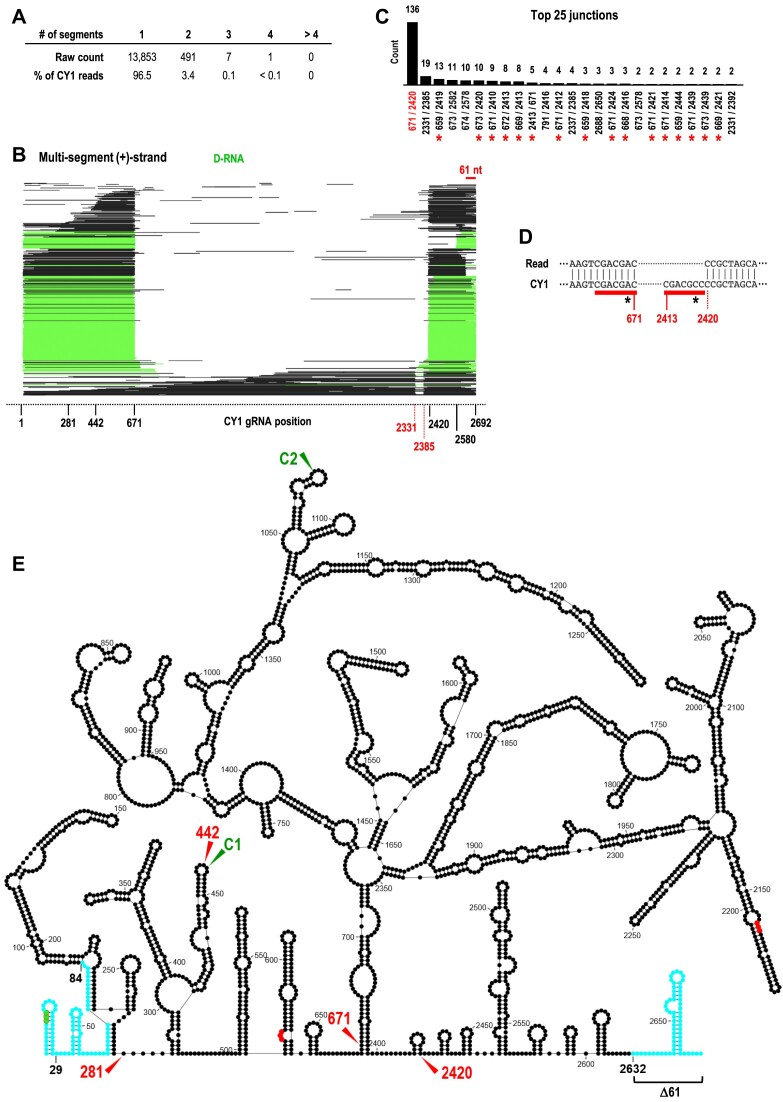
D-RNAs in the CY1 transcriptome. (**A**) Numbers of single and multi-segment reads in 6-wpi leaves. (**B**) Read alignment plot for 6-wpi leaf (+)-strand reads possessing multiple discontinuous segments. Reads are defined and colored as in Figure 2. Note that many reads have a truncation of the 3′ terminal 61 nt and that multi-segment, non-D-RNA reads have a common deletion of positions ∼2331–2385. (**C**) Counts of the top 25 discontinuous junction sites in reads. Close variants of the most abundant junction associated with D-RNAs (671/2420) are denoted with a red asterisk. (**D**) Sequence surrounding the 671/2420 junction compared to the reference CY1 gRNA sequence. Seven similar residues are found immediately adjacent to this junction in CY1 gRNA (underlined in red) that differ only at their sixth positions (asterisks). All reads containing junction 671/2420 have an ‘A’ at position 6 (asterisk) within the 7 nt stretch. (**E**) Secondary structure of CY1 showing locations of the junction sequences for the major D-RNA (671/2420), the 3′ terminal positions for F281 and F442, and cleavage sites C1 and C2. Commonly found truncations at the 5′end (between positions 29 and 84) and 3′end (∼61 nt) are in blue. Start codon is in green and stop codons are in red.

At 6-wpi, reads with two discontinuous segments comprised 3.5% of CY1 leaf reads with only 0.1% of CY1 leaf reads having three or more segments (Figure 3A). The read alignment plot of all multi-segment (+)-strand reads revealed that most corresponded to D-RNAs (i.e. possessing both 5′ and 3′ co-terminal segments) (Figure 3B, green), with a minor fraction corresponding to longer transcripts with a small deletion between positions 2331 and 2385. Of the top 25 junctions between discontinuous segments, 51% (136 of 265) contained position 671 joined to position 2420 (Figure 3C), representing a D-RNA containing positions 1 to 671 joined to positions 2420 to 2692. Of the remaining junctions, 31% were located proximal to 671 and 2420 and thus belonged to very similar D-RNA species (Figure 3C, red asterisks). Since the 5′ segment of the major D-RNA was identical to F671, this would also contribute to the substantial drop-off at this position in the coverage map (Supplementary Figure S2, DO3), as the drop-off would include both F671 reads and the 671 nt portion of most D-RNA reads.

Figure 3E shows the location of the major D-RNA junction sites as well as the 3′ends of F281 and F442 on the known secondary structure of CY1 (28). Note that the D-RNA junction sites (671 and 2420) are located near to each other within this structure. Inspection of sequences surrounding the D-RNA 671/2420 junction revealed 7 similar nt; 5′CGACGAC on the 5′ side and 5′CGACGCC on the 3′ side (single mismatch at position 6 is underlined) (Figure 3D). The presence of similar sequences neighboring the junction suggests that these D-RNAs were generated by an RdRp template switch, which are frequently associated with sequence repeats at the junctions (3). Since all reads containing the 671/2420 junction possessed an ‘A’ at position 6 within the 7 nt stretch, template switching occurred after transcription termination by the RdRp on the 5′ side of the junction. Therefore, we propose that F671 is generated during (+)-strand synthesis when the RdRp prematurely terminates transcription at position 671 and releases the nascent strand. Alternatively, the D-RNA is generated if the RdRp continues transcription at position 2420 before releasing the nascent strand.

### Minor (+)-strand transcripts

In addition to these major (+)-strand constituents, read alignment plots for 2- and 6-wpi leaves and roots contained a large number of subgenomic length fragments scattered throughout the length of the gRNA (Figure 2). In particular, the read alignment plot for 6-wpi leaves revealed two distinctive vertical ‘lines’ (labeled C1 and C2), suggesting a large number of reads were cleaved at or near these sites. Since DRS does not sequence the final ∼13 nt at the 5′ ends of RNA molecules, the 3′ends of fragments were used to locate the sites of cleavage. C1 (position 442) and C2 (position 1070) corresponded with the locations of prominent (+)-strand coverage map grooves Gr2 and Gr5 (Supplementary Figure S2), and neither corresponded to junctions in recombinant multi-segment reads (Figure 3A). Therefore, C1 and C2 must be the result of individual reads terminating and beginning at/near positions 442 and 1070, respectively, rather than recombinant reads containing small deletions (Supplementary Figure S3). Cleavage at C1 would generate F442, strongly suggesting that F442, unlike F671, is cleavage derived. Furthermore, the lack of cleavage site associated with F281 suggests that an alternative mechanism is responsible for generation of the smaller lncRNA.

To determine if C1 and C2 reflect cleavage sites generated by major in-line cleavage events [caused by nucleophilic attack of the 2′OH on the phosphate backbone of flexible residues (56)], we conducted DRS on CY1 gRNA IVT subjected to conditions that promote in-line cleavages (Supplementary Figure S4). Unlike the IVT reads, the in-line cleavage reads showed a 3′ skew of 104%, likely due to RNA auto-hydrolysis resulting in 3′ends that lack a 3′ hydroxyl and, therefore, are not directly amenable to DRS (56). The read alignment plot and coverage map for CY1 gRNA IVT subjected to these conditions did not exhibit cleavage sites or grooves, suggesting that autocatalytic digestion is not responsible for C1 and C2. Interestingly, both C1 and C2 map to the terminal loops of hairpins in highly structured regions that are structurally conserved in all Group2/Class2 ULVs (Figure 3E).

### (+)-strand transcripts missing 5′ and/or 3′ terminal sequences

gRNAs containing truncations of 5′ or 3′ terminal sequences have been previously reported in (+)-strand RNA virus infections but their function, if any, remains unknown (9). Curiously, 14.2% of the (+)-strand CY1 reads in 2- and 6-wpi leaves, and 11.4% of the (+)-strand reads in 6-wpi roots were missing the 3′ terminal 61 nt (positions 2632–2692), suggesting that this location represents an additional cleavage site (Figures 2 and 3E). Coincidentally, residues at positions 2632–2638 are involved in a long distance interaction with the apical loop of the –1 frameshifting recoding structure element located just downstream of the p21 ORF stop codon (57). One possible reason for the generation of these truncated transcripts is if this critical long-distance interaction required for efficient frameshifting occurs more optimally *in trans* than *in cis*. Additionally, within these 61 nt are the 3′ terminal GCCC-OH motif and the 3′ proximal Pr hairpin, which are elements promoting (–)-strand synthesis in many viruses in the *Tombusviridae* (58,59). Therefore, gRNAs containing this truncation could be templates for multiple rounds of translation of the RdRp without being templates for replication, which would require full-length gRNAs.

Additionally, many transcripts were missing 5′ terminal sequences with 5′end points spanning between positions 29 to 84 (Supplementary Figure S2A; Figure 3E). The start codon for the p21 ORF is located at position 9, and 5′ proximal elements enhancing viral translation were mapped within the 5′ terminal 33 nt (44). This suggests that transcripts missing 5′ terminal sequences may not be viable templates for replication or efficient translation of a truncated ORF1.

### (–)-Strand transcripts and foldback RNAs

**Figure 4.**
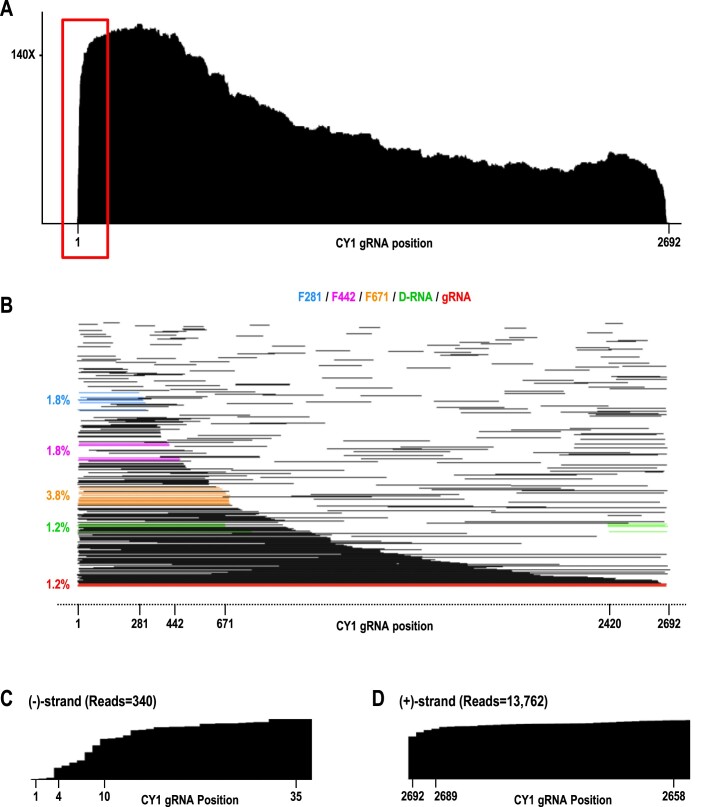
Coverage map and read alignment plot of single-stranded (–)-strands in 6-wpi leaves. (**A**) Coverage map. (**B**) Read alignment plot. Reads corresponding to full-length gRNA, D-RNA, F281, F442 and F671 fragments are colored as in Figure 2, with (–)-strand read 3′end positions permitted to be somewhat less precise than for (+)-strands (allowed to be within 30 nt rather than only 10 nt of their target RNA 3′end), due to the lower precision of (–)-strand 3′end sequences. Numbering of CY1 nucleotides is for the corresponding (+)-strand. (**C**) Enlargement of the boxed region from A showing the missing 3′ terminal CCC in all (–)-strand reads that cover this location. (**D**) Comparable region from 6-wpi (+)-strand reads that covers the 3′ terminal region (3′ terminal sequence is also CCC-OH), showing no loss of reciprocal nucleotides. X-axis was flipped to aid in comparison with (–)-strand reads.

CY1 (–)-strand sequences were present in reads containing only (–)-strand sequence, as well as in reads that were (±)-strand hybrid foldbacks. Reads containing only (–)-strand were 2.4% of 6-wpi leaf CY1 reads. Cleavage maps for these reads contained no distinctive grooves or drop-offs, and read alignment plots had no major cleavage sites (Figure 4A and B). Full-length (–)-strand reads were 1.2% of the total (–)-strand reads at 6-wpi, similar to the (–)-strand reads for D-RNAs, and less than the 3.8% reads for F671. This suggests that D-RNA, and possibly F671, may be templates for replication. Although a few (–)-strand reads were near complements of F281 and F442, it is not clear whether these were simply spurious (–)-strand fragments or reflected synthesis of the complementary strand. Curiously, virtually all (–)-strand-only reads that extended to near the 5′end of (+)-strand gRNA were missing their 3′ terminal three cytidylate residues. This implies that transcription of full-length (–)-sense transcripts terminated at least three residues early rather than extending beyond the uridylate (underlined) at the 5′end of the (+)-strand template (5′GGGUAAA-OH) (Figure 4C). If full-length (–)-strands are indeed missing three cytidylates at their 3′ends in infected plants, then the three guanylates at the 5′ends of (+)-strands would require non-templated addition. In contrast, 3′ terminal coverage of full-length (+)-strands (including their own 5′CCC-OH motif) was consistently high (Figure 4D).

**Figure 5.**
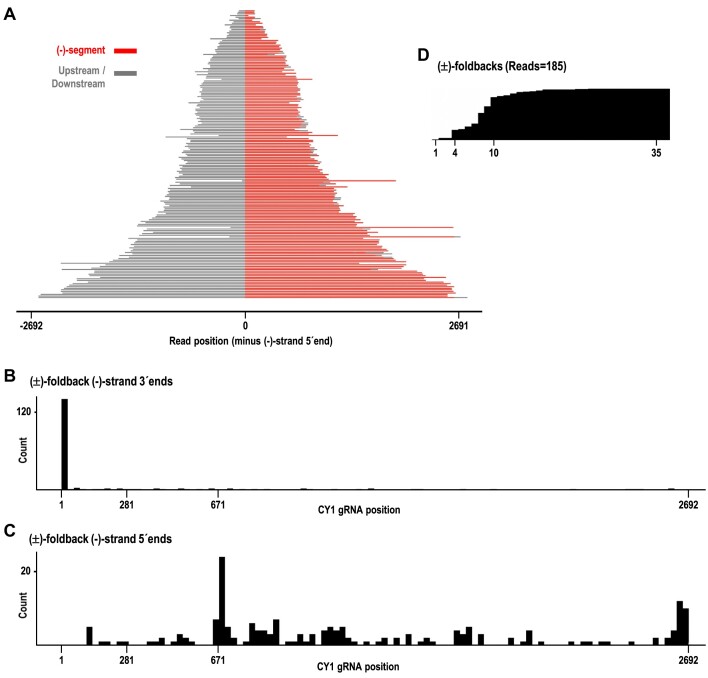
(±)-foldback RNAs identified in the 6-wpi leaf sample. (**A**) Plot of all (±)-foldback reads. (–)-strand segments are colored red and upstream sequences (containing the (+)-strand segments) are colored gray. Sequence downstream of the (–)-strand segment that did not align to CY1 is also colored gray. (**B**) The distribution of 3′ terminal positions for the (–)-strand sequences within foldbacks. (**C**) The distribution of 5′ terminal positions for the (–)-strand sequences within foldbacks. (**D**) Partial coverage map showing the missing 3′CCC at the 3′end of nearly all foldback read (–)-strand sequences.

Reads containing (–)-strand sequence downstream of (+)-strand sequence (foldbacks) comprised 31.4% of all reads containing (–)-strand sequence (Figure 5A). Identification of foldback RNAs using DRS was complicated by the propensity of DRS to misread or skip residues upstream of long hairpin structures (manuscript in preparation); thus foldbacks were identified by: (i) their general symmetry of length between the upstream sequence and (–)-strand sequence; (ii) hairpin-like structures when folded *in silico* and (iii) dot plot analysis (Figure 5A and Supplementary Figure S5). Past studies of poliovirus-infected HeLa cells reported similar levels of foldback molecules (25–50% of isolated dsRNAs) (19). No (–/+) foldbacks were detected, and nearly all foldbacks contained (–)-sense segments extended to near the 5′end of the complementary (+)-strand gRNA (Figure 5B). Since foldbacks are generated by the viral RdRp, the lack of foldbacks associated with interior CY1 fragments suggests that these fragments arose after CY1 replication.

Foldback (–)-strand segments had 5′ends located primarily at position 671 or near the CY1 3′ terminus (i.e. these latter foldbacks were of full-length CY1 gRNA) (Figure 5C). Since foldbacks are generated by viral RdRp re-initiating transcription before releasing the nascent strand (full-length or prematurely-terminated), this supports the genesis of F671 by the RdRp. In contrast, the lack of foldback reads corresponding to F281 and F442 suggests that neither are generated by premature termination by the viral RdRp. Since F281 is also not associated with a prominent cleavage site as is F442, it may have been generated by degradation of larger viral transcript(s) by a 3′-to-5′ exonuclease. As with the non-hybrid full-length (–)-sense reads, foldbacks were missing at least their 3′ three cytidylates (Figure 5D) Intriguingly, a large number of (±)-foldback reads possessed (–)-strand sequence in their foldback loop and/or partially down their 5′ side, without a corresponding (+)-strand template for transcription of these (–)-strand sequences (Supplementary Figure S6). Some (±)-foldback reads also possessed non-viral sequences in their foldback loop regions, possibly arising from templateless elongation by the viral RdRp (60), and further suggesting that additional, currently unknown mechanisms for foldback generation may exist. In addition, the relatively high abundance of (±)-foldbacks among reads containing (–)-strand sequence suggests that care should be taken when identifying viral replication intermediates, since (±)-foldback hairpins would bind to dsRNA antibodies, which are commonly used to identify ‘dsRNA’ viral replication intermediates (61–65).

### The CY2 transcriptome in agroinfiltrated *N. benthamiana* and natural field plants

We next investigated how the viral transcriptome in agroinfiltrated *N. benthamiana* compares with the viral transcriptome in a natural host infection in a field setting. Since CY1 has only been found once in infected citrus (28,29), we examined plants infected with closely related CY2, which differs from CY1 by expressing the ORF5 protein from an sgRNA (Figure 1A). Since *C. sativa* is a perennial plant, and the sampled plant was likely in a later-stage of infection, we used a 14-wpi time point in *N. benthamiana* to compare with the infected field sample. Only leaf transcriptomes were examined, as root samples were not available from the grower.

**Figure 6.**
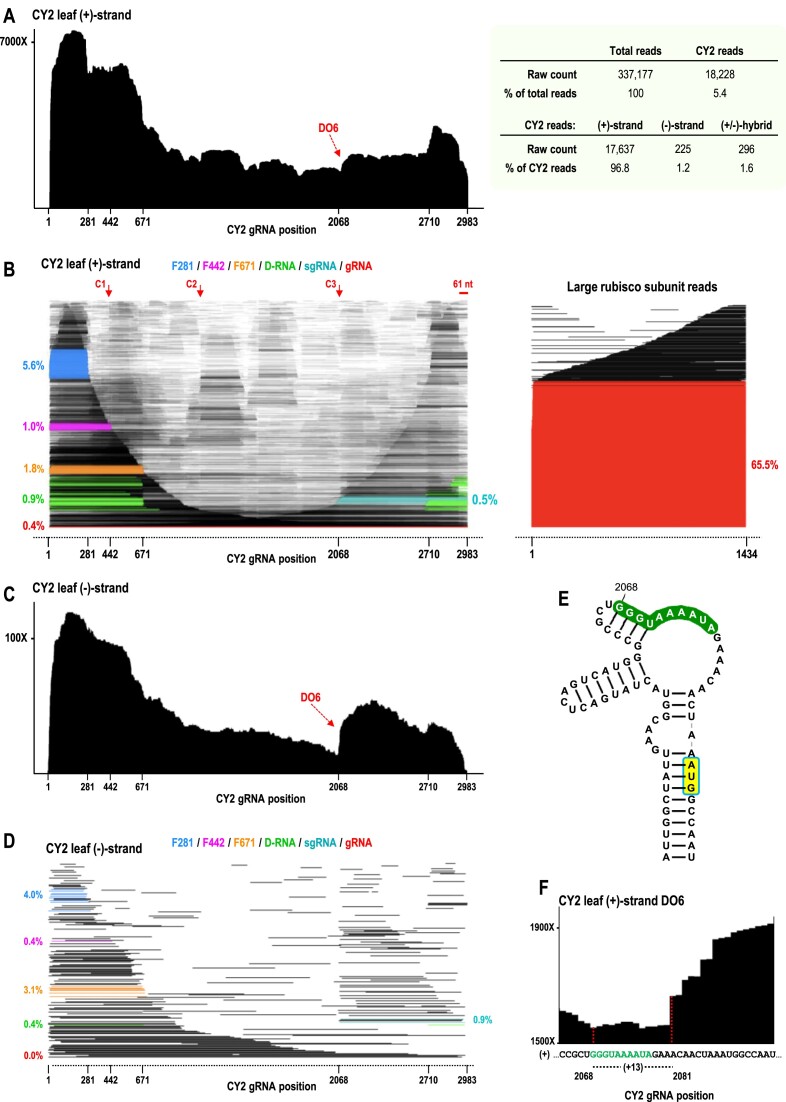
The CY2 transcriptome in 14-wpi *N. benthamiana* leaves. (**A**) Left, (+)-strand coverage map showing a new drop-off (DO6) not found for CY1. Right, DRS read summary. (**B**) Left, (+)-strand read alignment plot. Right, read alignment plot for rubisco from the same sample. Coloring is as shown in Figure 2 and sgRNA is colored aqua. CY2 exhibits an additional cleavage site (C3) compared with CY1, which is near the sgRNA start site. (**C**) Coverage map of (–)-strand reads showing new DO6 is also found for complementary strands. (**D**) Read alignment plot for (–)-strand reads. (**E**) Sequence/structure of the previously proposed sgRNA promoter. The carmovirus consensus sequence beginning at position 2068 is in green and the start codon of ORF5 is shaded yellow. (**F**) Closeup view of (+)-strand coverage map DO6. DO6 terminates at position 2081, which is 13 nt upstream of position 2068, coinciding with the tendency of DRS sequencing to terminate prematurely ∼13 nt away from the 5′ends of RNA molecules in general. The aqua-colored fragments in B and D are therefore identified as the CY2 sgRNA.

DRS sequencing data for CY2-infected *N. benthamiana* leaves at 14-wpi were similar to those for CY1 at 6-wpi in many aspects including: (i) CY2 reads were a similar 5.4% of total reads (Figure 6A, right); (ii) the (+)-strand coverage map displayed a large 5′ skew of 355%, as well as less pronounced 3′ skew of 112% (Figure 6A, left); (iii) 5′ co-terminal fragments F281, F442 and F671 were present, although F442 levels were lower for CY2 (Figure 6B); (iv) many (+)-strand reads contained the 61 nt truncation at the 3′end and (v) virtually all (–)-strand reads and (–)-strand portions of (±)-foldback reads were missing 3′ terminal sequences including the 5′CCC-OH motif.

**Figure 7.**
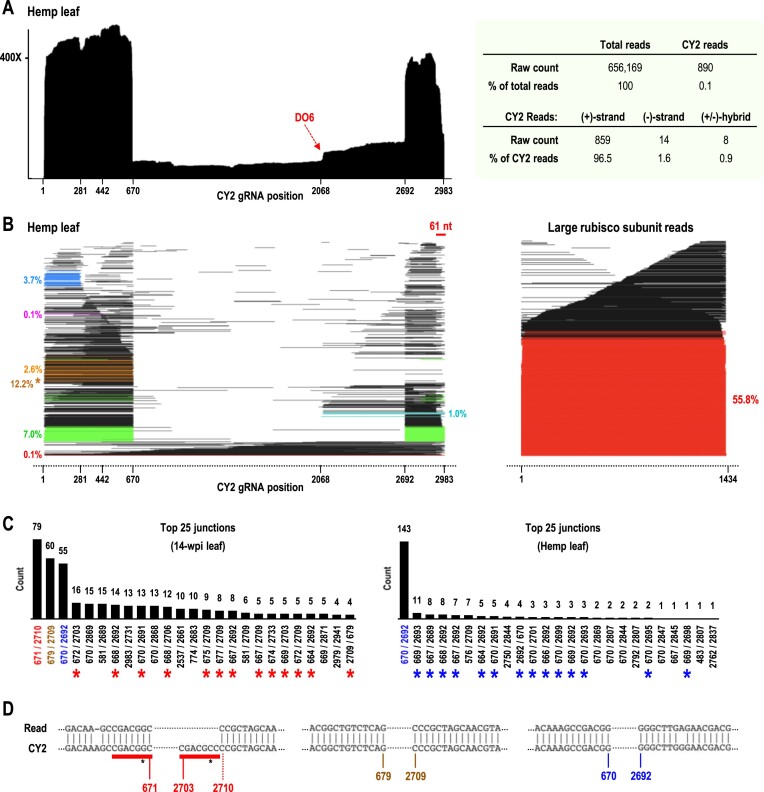
DRS of CY2 in infected *C. sativa* from Washington State. (**A**) Left, (+)-strand coverage map. Right, DRS read summary. (**B**) Left, (+)-strand read alignment plot. Right, read alignment plot for rubisco from the same sample. Coloring is as shown in Figure 6B. The 3′ segments of many D-RNA reads did not align to the viral genome (due to sequence differences), necessitating that F671 reads be required to have an overall read length within 60 nt of 671, in order to more accurately count F671 reads. Without this additional requirement, the F671 read count would be much higher at 12.2% (brown asterisk). (**C**) Counts of the top 25 most abundant junctions sequenced for 14-wpi leaf sample (left) and *C. sativa* sample (right). The 3 major D-RNA junctions found in *N. benthamiana* (left) and single major D-RNA junction found in *C. sativa* (right) are color coded, and other D-RNA junctions similar to these three are marked with red or blue asterisks. (**D**) CY2 genome alignments for the three major D-RNA junctions 671/2710, 679/2709, and 670/2692.

In contrast with 6-wpi CY1, CY2 had a lower percentage of full-length (+)-strand reads (0.4%) (Figure 6B). In addition, CY2 had fewer (–)-strand reads than CY1 (1.2% compared with 2.4%), and a higher percentage of (±)-foldbacks among reads containing (–)-strand sequence (46.9% compared with 31.4%). CY2 also had an additional drop-off (DO6) in both (+)- and (–)-strand coverage maps (Figure 6A and C), corresponding with the sgRNA start site that had been previously postulated based on the presence of a motif known as the carmovirus consensus sequence (GGG A/U_5-9_) (Figure 6E), which is found at the 5′ends of carmovirus and umbravirus sgRNAs (30). A sgRNA ‘imprint’ was also observed in the (–)-strand read alignment plot (Figure 6D). The 5′ends of the (+)-strand reads, which for DRS normally terminate ∼13 nt short of the true 5′end, correlated precisely with the site previously hypothesized to be the 5′end of the CY2 sgRNA (Figure 6E and F) (30). This previous determination was based on the presence of a motif known as the carmovirus consensus sequence (GGG A/U_5-9_) (Figure 6E), which is found at the 5′ends of carmovirus and umbravirus sgRNAs. Unlike CY1, CY2-infected *N. benthamiana* leaves contained three major D-RNAs with the following junctions: 671/2710, which corresponded to major CY1 D-RNA junction 671/2420 (CY2 has two segments not present in CY1), 679/2709 and 670/2692 (Figure 7C and D).

DRS of the CY2-infected *C. sativa* leaf sample revealed that viral read levels were much lower than found for 14-wpi *N. benthamiana* leaves (0.1% compared with 5.4% of total reads, respectively; Figure 7A, right). Additionally, the *C. sativa* leaf sample exhibited a higher percentage of (–)-strand reads (1.6% versus 1.2%) and lower percentage of (±)-hybrid reads (0.9% versus 1.6%). The (+)-strand coverage map for the *C. sativa* leaf sample exhibited dramatic 5′ and 3′ skews of 960% and 767%, respectively (Figure 7A), with sharp intervening drop-offs centered at positions 670 and 2692. The read alignment plot revealed that these sharp drop-offs corresponded to the presence of a single major D-RNA with a junction at 670/2692 (Figure 7B and C, right), which was one of the three major D-RNAs in 14-wpi *N. benthamiana*. This finding suggests that this D-RNA is more fit than the other two D-RNAs that arose de novo in *N. benthamiana*. Levels of the three lncRNAs in the *C. sativa* sample also differed from those in the *N. benthamiana* sample: F671 (2.6% compared with 1.8%); F442 (0.1% compared with 1%); and F281 (3.7% compared with 5.6%).

### DRS results are not quantifiable

**Figure 8.**
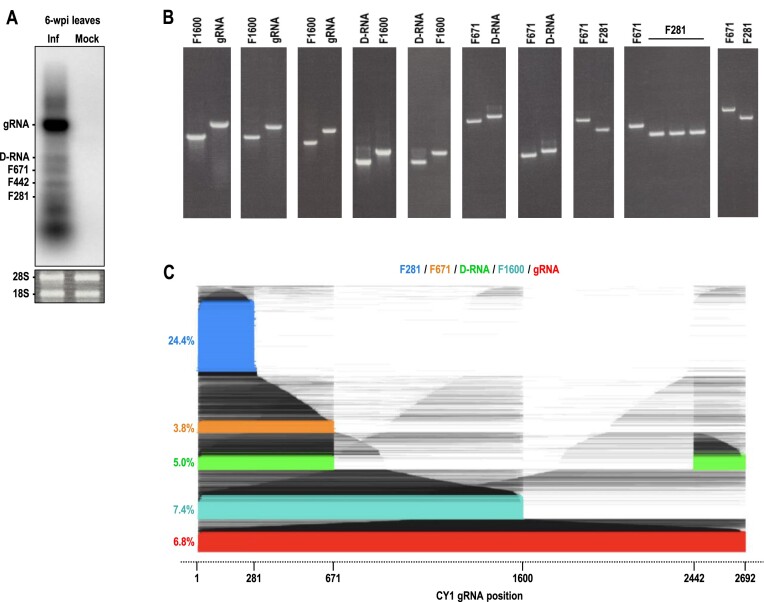
DRS of IVT size marker RNAs. (**A**) Northern blot of total RNA extracted from 6-wpi *N. benthamiana* leaves infected with CY1. Radiolabeled probes were complementary to positions 90 to 265. Bands were identified as migrating to the same positions as size markers included in the same gel. (**B**) Ethidium bromide-stained agarose gels used to normalize IVT size marker RNAs. F281, F671, D-RNA (922 nt), F1600 and gRNA (2692 nt) were *in vitro* transcribed, normalized according to band intensity, diluted according to RNA length, mixed in equimolar amounts and then sequenced by DRS. (**C**) Read alignment plot for all (+)-strand reads, with full-length RNAs color-coded and percentage counts shown. Note that the D-RNA sequence used in this experiment contains junction 671/2442 and is different from the predominant D-RNA observed for 6-wpi leaf (junction 671/2420).

The unexpectedly low levels of full-length gRNA reads in all of the DRS-generated transcriptome data sets prompted the examination of major transcripts in CY1-infected *N. benthamiana* leaves at 6-wpi by Northern blot using probes targeting positions 90 to 265, which should hybridize equally to (+)-strand gRNA, D-RNA and the lncRNAs (Figure 8A). According to the Northern blot, gRNA was much more abundant than D-RNA, F671, F442 and F281, strongly suggesting that DRS does not equally capture viral RNAs. One possibility is that DRS overestimates shorter reads, with read abundance directly related to read length. To test for the efficiency of DRS in sequencing CY1 RNAs of different sizes, a control experiment was conducted on a sample containing equimolar amounts of *in vitro* transcribed F281, F671, D-RNA, full-length gRNA, as well as a 5′ co-terminal 1600 nt fragment (F1600) that was not found *in planta* (Figure 8B). DRS results for this sample revealed that full-length F281 IVT RNA was overrepresented compared with the other IVT RNAs (Figure 8C). However, there was no correlation between sequencing efficiency and size for the other IVT RNAs (Figure 8C), suggesting that the failure of DRS to accurately capture full-length gRNA extracted from plants was not due to a simple size bias. This result implies that DRS may not accurately quantify different transcripts within a sample although relative levels of identical transcripts between samples should still be meaningful.

### Concluding remarks

The ability of nanopore sequencing to generate reads corresponding to the near full-length sequence of individual viral transcripts for the first time allows a snapshot of the complete transcriptome of an RNA virus. It is important, however, to understand the limitations of this technique. Since sequencing requires that the RNA be polyadenylated, the abundance of an RNA may be biased due to structural resistance and/or propensity for adenylation. Additionally, DRS generates non-random errors, including a substantial amount of incorrect base calling and skipping of sequential residues upstream of large hairpin-like structures (e.g. foldbacks) or other highly structured regions, causing the alignment/identification of these viral RNA sequences to fail, and leading to their mislabeling as non-viral (manuscript in preparation).

Despite these limitations, DRS of CY1- and CY2-infected leaves and roots revealed a substantial amount of novel information including three 5′ co-terminal viral RNAs (F281, F442, F671) that are apparently generated by different mechanisms (i.e. possible 3′-to-5′ degradation of larger viral transcripts, endonucleolytic cleavage, and premature termination of (+)-strand synthesis, respectively). The absence of the 3′ cap independent translation enhancer required for efficient translation of full-length CY1 gRNA (44) suggests that F281, F442 and F671 may not be serving as templates for protein translation, and thus may be novel 5′-co-terminal lncRNAs. Among (+)-strand RNA plant viruses, 5′ co-terminal lncRNAs have only been reported for CTV, where they function in overcoming plant defenses (13,14). Whether these abundant CY1 and CY2 lncRNAs also play roles in the virus infection cycle awaits further investigation. DRS also revealed the presence of two specific cleavage sites in apical loops of highly conserved structures, which, in addition to F442, give rise to large amounts of minor subgenomic-length internal fragments, particularly for leaf samples at later stages of infection (Figure 2). Whether these cleavage sites are related to host defenses targeting the gRNA for degradation is not known. The lack of 3′ terminal residues in (–)-strand reads and 61 nt truncations at the 3′ ends of many (+)-strand gRNA reads suggests additional modes of replication and translation may exist for CY1 and CY2. DRS of other RNA viruses will be necessary to determine if these results are unique to ULVs or representative of other plant (+)-sense RNA viruses.

## Supplementary Material

lqae104_Supplemental_File

## Data Availability

All sequencing data .fastq files of basecalled reads can be accessed using the following NCBI SRA IDs: 2-wpi CY1-infected *N. benthamiana* leaf: SRX24732504 2-wpi CY1-infected *N. benthamiana* root: SRX24732505 6-wpi CY1-infected *N. benthamiana* leaf: SRX24732506 6-wpi CY1-infected *N. benthamiana* root: SRX24732507 14-wpi CY2-infected *N. benthamiana* leaf: SRX24732508 CY2-infected hemp leaf field sample: SRX24732509 IVT CY1 gRNA: SRX24732510 IVT CY1 gRNA in-line probing: SRX24732511 IVT RNA size markers (with high-accuracy basecalling): SRX25400331 IVT RNA size markers (with fast basecalling): SRX24732512 All BLAST alignment JSON outputs to viral genome sequences can be accessed using the following figshare IDs: 2-wpi CY1-infected *N. benthamiana* leaf: 26 343 598 2-wpi CY1-infected *N. benthamiana* root: 26 343 601 6-wpi CY1-infected *N. benthamiana* leaf: 26 343 604 6-wpi CY1-infected *N. benthamiana* root: 26 343 610 14-wpi CY2-infected *N. benthamiana* leaf: 26 343 613 CY2-infected hemp leaf field sample: 26 343 622 IVT CY1 gRNA: 26 343 655 IVT CY1 gRNA in-line probing: 26 343 679 IVT RNA size markers (with high-accuracy basecalling): 26 343 691 IVT RNA size markers (with fast basecalling): 26 343 700 All BLAST alignment JSON outputs to host large rubisco mRNA can be accessed using the following figshare IDs: 2-wpi CY1-infected *N. benthamiana* leaf: 26 343 712 2-wpi CY1-infected *N. benthamiana* root: 26 343 715 6-wpi CY1-infected *N. benthamiana* leaf: 26 343 718 6-wpi CY1-infected *N. benthamiana* root: 26 343 721 14-wpi CY2-infected *N. benthamiana* leaf: 26 343 724 CY2-infected hemp leaf field sample: 26 343 727 All custom Python scripts created for this report have been deposited in the following GitHub repository: https://github.com/pzhaojohnson/nar-gab.johnson-etal. All data generated for this report and all custom Python scripts have been further bundled together at the following figshare link: https://doi.org/10.6084/m9.figshare.25810258.v1.
