# Supplementary material for: Direct nanopore RNA sequencing of umbra-like virus-infected plants reveals long non-coding RNAs, specific cleavage sites, D-RNAs, foldback RNAs, and temporal- and tissue-specific profiles
Source: NAR Genom Bioinform. 2024 Aug 16;6(3):lqae104. doi: 10.1093/nargab/lqae104 (PMC11327873; doi:10.1093/nargab/lqae104)
Supplement: lqae104_Supplemental_File [file lqae104_supplemental_file.pdf]

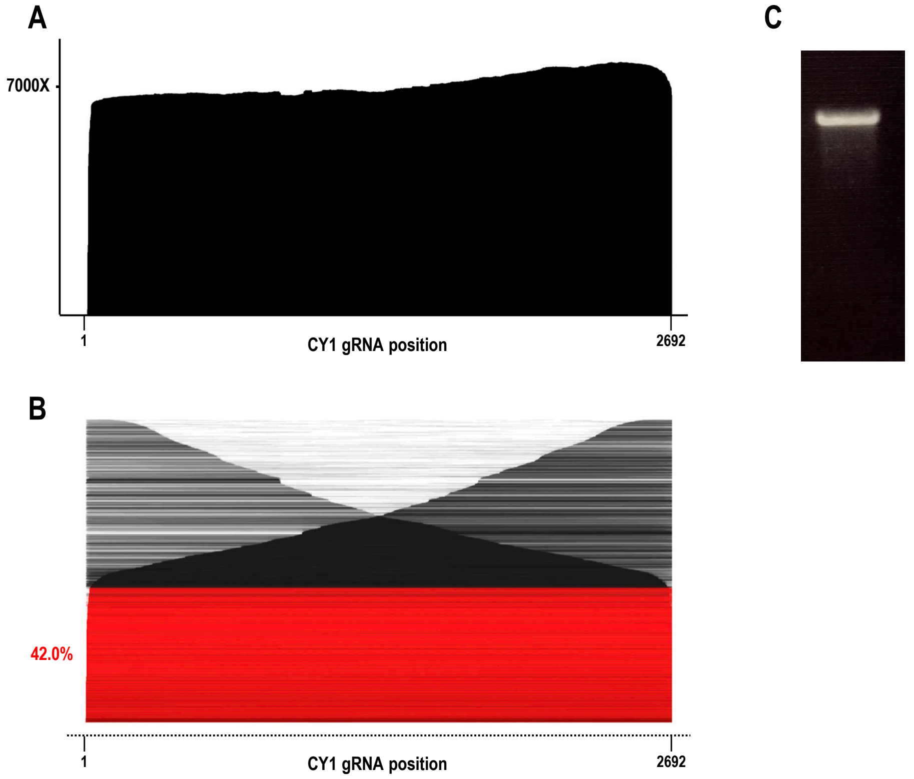

Figure S1. (+)-strand coverage map and read alignments plot for IVT CY1 gRNA. (**A**) Coverage map for the CY1 IVT RNA (reproduced here from Figure 1B). (**B**) Read alignment plot showing every read at a discrete position on the Y-axis and arranged according to aligned length. Full-length gRNA reads are colored red and the percentage of full-length reads among all CY1 IVT reads is shown. Full-length gRNA reads are defined as having a single continuous segment, a 5´end position within 30 nt of position 1 of the viral genome, and a 3´end position within 10 nt of the 3´end of the viral genome (i.e., position 2692). 5´ terminal positions were allowed to be less precise due to the lower precision of 5´end sequencing by DRS (the last portion sequenced), which can affect where the CY1 alignment ends. (**C**) Ethidium bromide-stained agarose gel of IVT CY1 input RNA for the DRS run.

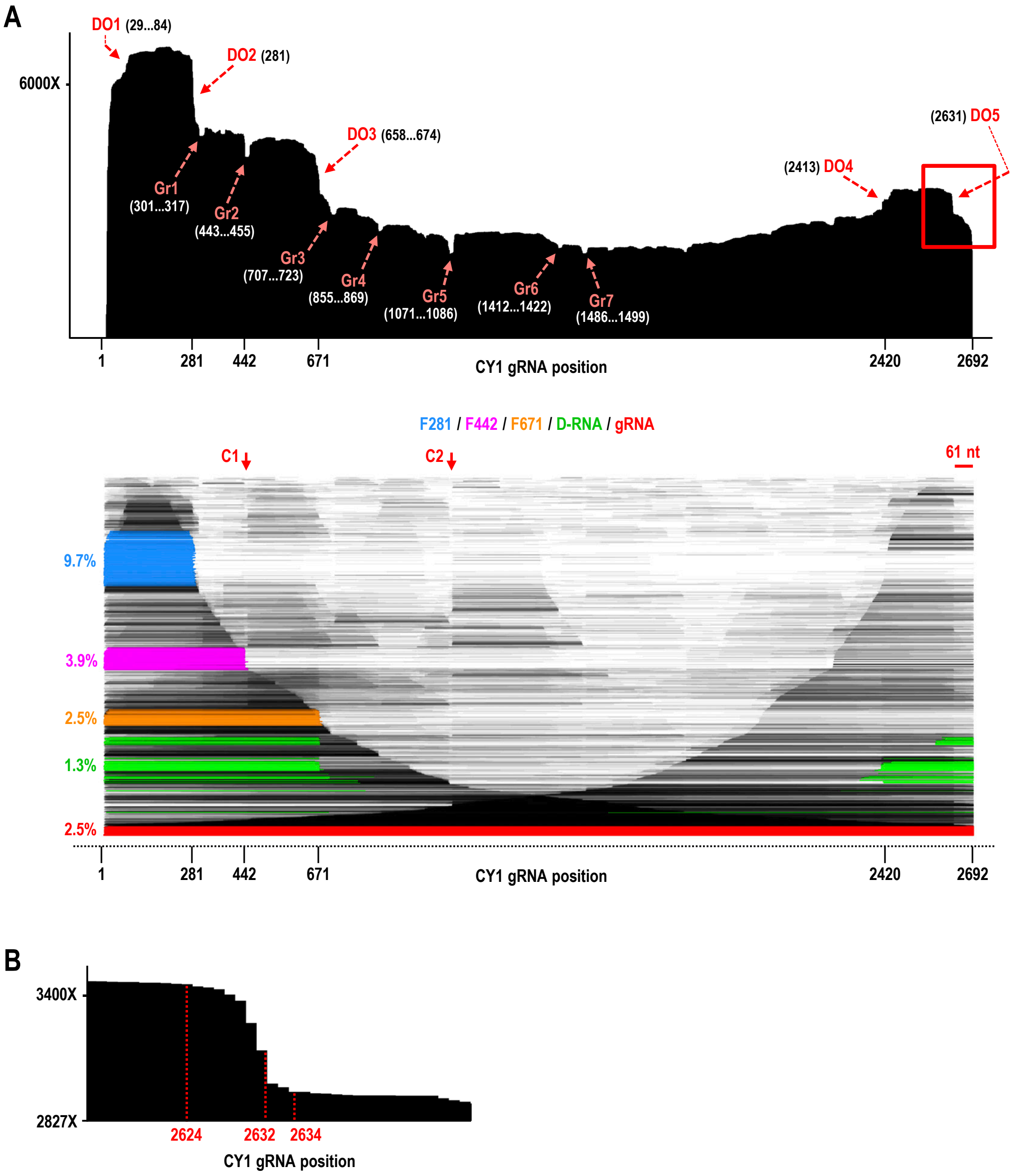

Figure S2. Comparison of coverage map and read alignments plot for CY1 (+)-strands from 6-wpi leaves. (**A**) Top, enlarged coverage map. Major drop-offs (labeled DO1 through DO5) and grooves (labeled Gr1 through Gr7) are denoted. Approximate positions for drop-offs and grooves are given in parentheses. Groove positions indicate the 5´- and 3´-most positions inside the groove. Bottom, enlarged read alignments plot. Locations where a large number of CY1 fragments both terminate and begin are denoted by arrows labeled C1 (corresponds to Gr2) and C2 (corresponds to Gr5). Location of the 3´ terminal 61 nt (corresponds to DO5), which are missing in many (+)-strand transcripts containing otherwise 3´ proximal sequences, is also labeled and likely represents an additional cleavage site. Major (+)-strand RNA reads are color coded. (**B**) Enlargement of the boxed region in A showing missing 3´ terminal sequence centered around 61 nt from the 3´end.

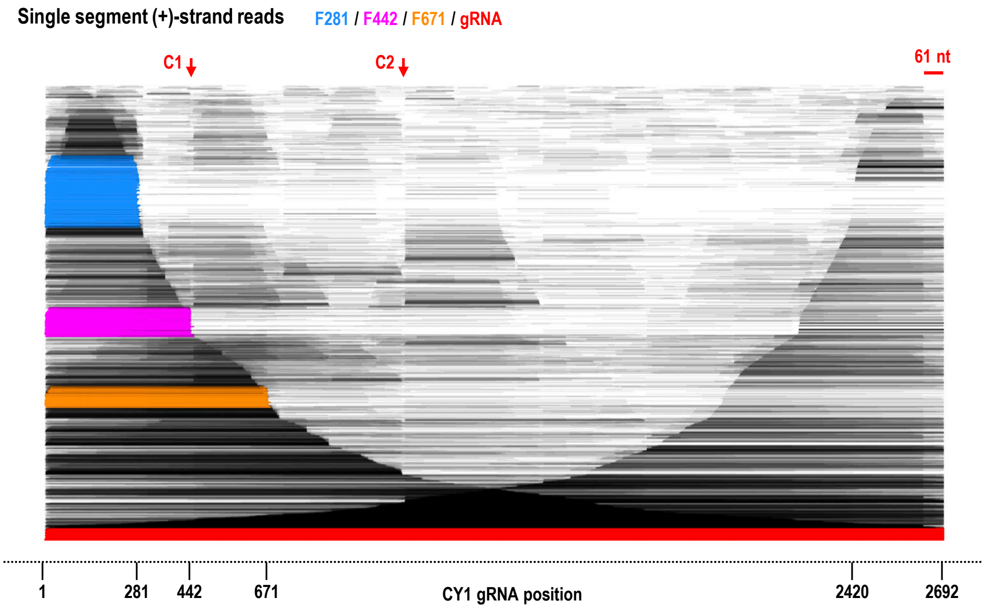

Figure S3. Read alignments plot of single segment (+)-strand reads for CY1 6-wpi leaves. Prominent cleavages C1 and C2 are only found in the single segment read alignment plot, indicating that they do not arise from deletions within discontinuous multi-segment reads.

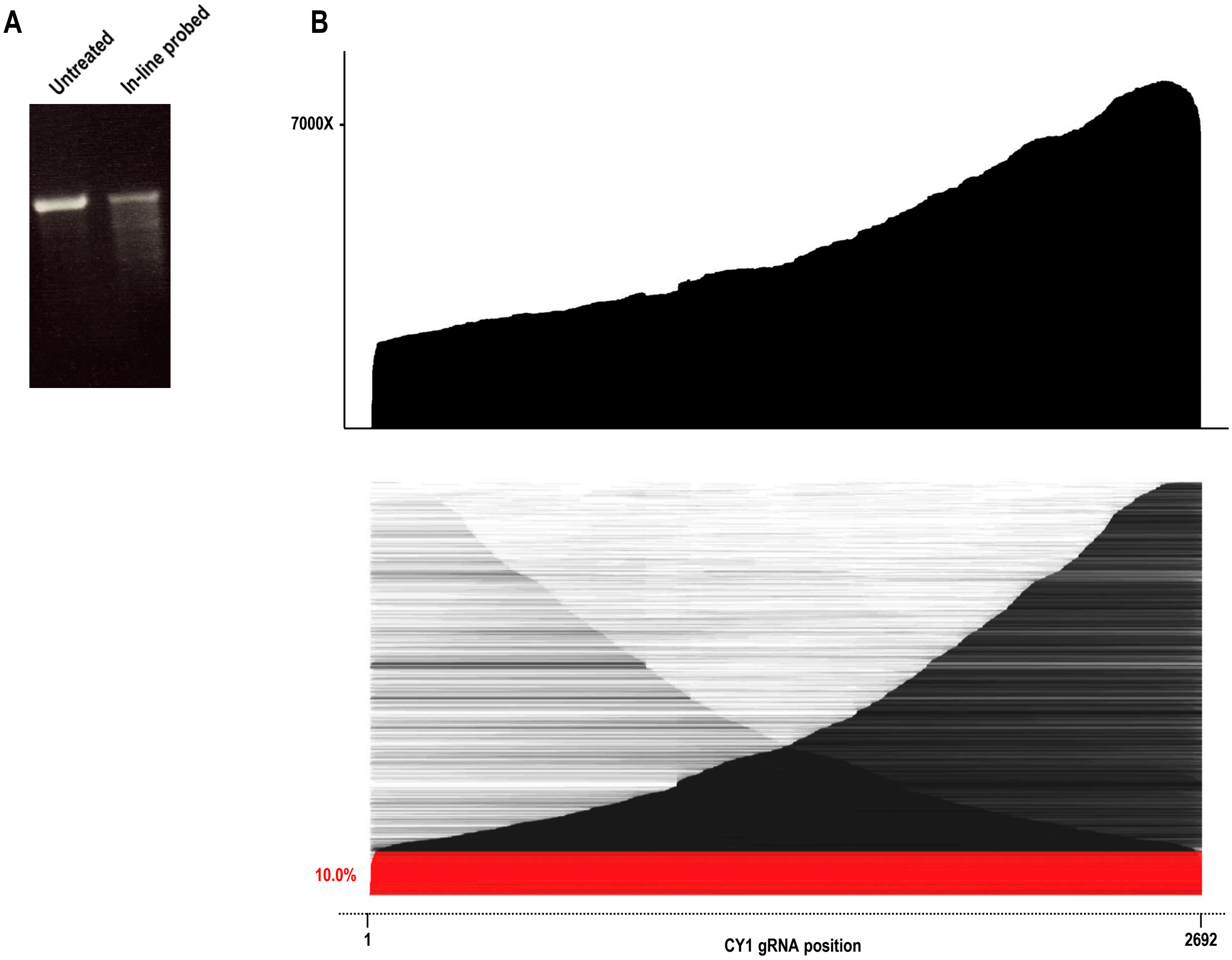

Figure S4. Coverage map and read alignments plot for IVT CY1 gRNA subjected to limited in-line cleavage do not contain distinctive grooves or cleavage sites. (**A**) Ethidium bromide-stained agarose gel of the IVT CY1 gRNA before (untreated) and after in-line probing. (**B**) Coverage map (top) and read alignments plot (bottom) obtained for IVT CY1 gRNA subjected to in-line probing. Reads corresponding to full-length gRNA are colored red and are defined as in the legend to Figure 2. The 3´ skew in the coverage map likely results from the lack of a 3´OH on 5´ fragments after autocleavage, which would not be sequenced by DRS. These 5´ fragments may eventually gain a 3´OH through hydrolysis of their 2´,3´-cyclic phosphate group, possibly explaining the relatively small number of 5´ co-terminal fragments.

A

# of segments	2	3	4	>4
Raw count	80	6	1	0
% of alignable (±)-hybrids	92.0	6.9	1.2	0

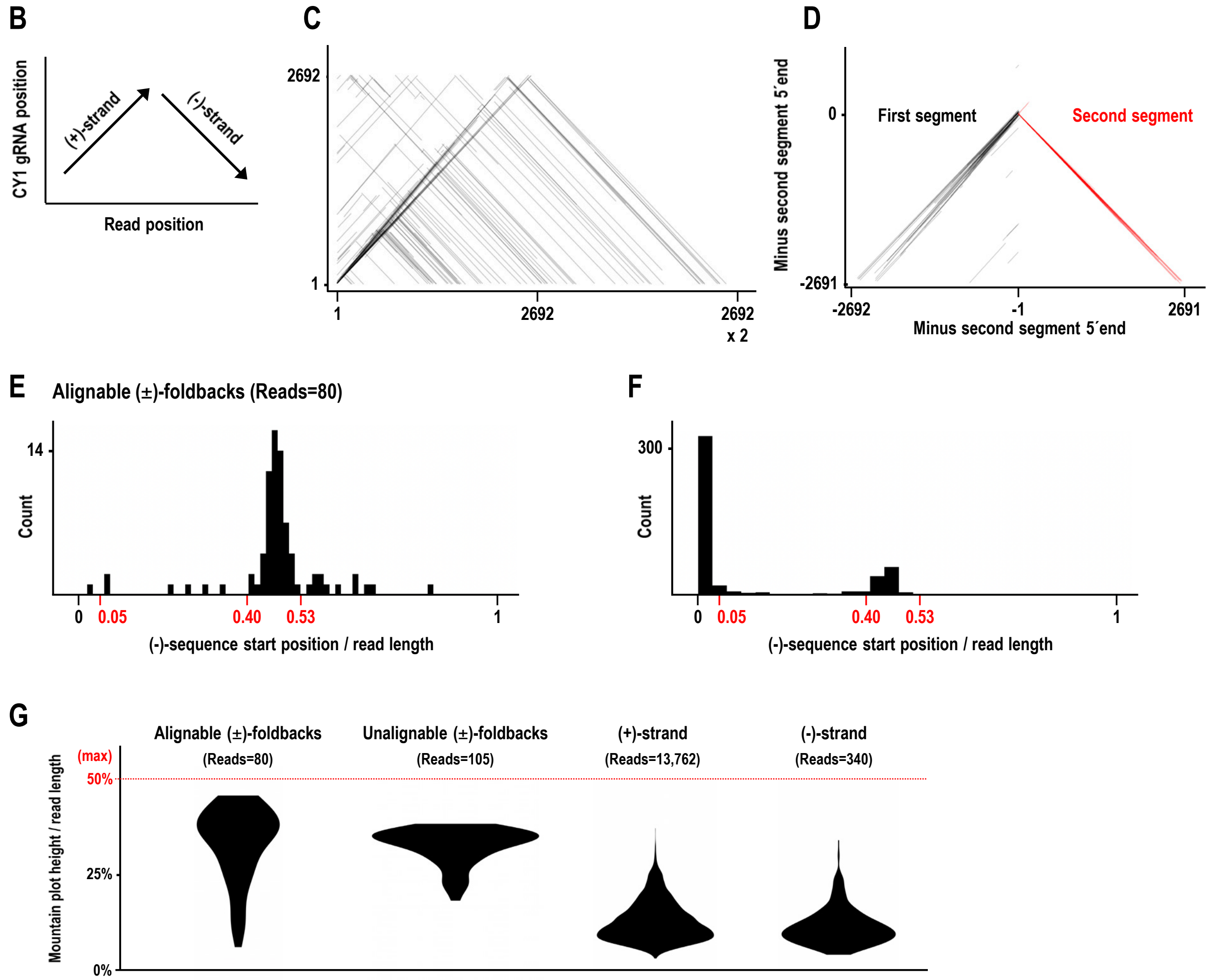

Figure S5. Identification of foldback RNA reads for CY1 6-wpi leaf. (**A**) Read counts grouped by the number of aligned segments present in reads among the 87 (±)-strand hybrid reads for which (+)-strand segment(s) were detected by sequence alignment using BLAST+ (termed the alignable (±)-hybrids). Due to sequencing errors committed by DRS for the upstream sides of long hairpin structures (such as foldback RNAs) the (+)-strand segments of many (+/-)-hybrid reads were not detected by sequence alignment. (**B**) Dot plot in which positions in a read were plotted against their corresponding aligned position in the viral genome. This results in (+)-strand sequences going from bottom-left to top-right, and (-)-strand sequences going from top-left to bottom-right. (**C**) Dot plot of the 87 alignable (±)-hybrid reads. (**D**) Dot plot of the 80 alignable (±)-hybrid reads possessing exactly two segments, with X and Y axes normalized according to the 5´end of the second segment present in each read. First and second segments also colored black and red, respectively, for each read. With this normalization, these 80 (±)-hybrid reads almost all perfectly overlap, and were termed the alignable (±)-foldback reads. (**E**) Histogram of the ratio of the starting position of (-)-strand sequence in each read divided by the read length for the 80 alignable (±)-foldback reads. (**F**) Histogram of the same ratio as in E but for the 503 reads for which (-)-strand sequence was detected by sequence alignment but for which no (+)-strand sequence was detected by sequence alignment. 340 reads had a ratio ≤ 0.05, indicating minimal upstream sequence, and were true (-)-strand reads. 105 reads had a ratio between 0.4 and 0.53, inclusive, indicating upstream sequence of comparable length to the downstream (-)-strand sequence present in each read, and were termed unalignable (±)-foldback reads. (**G**) Distributions of the normalized mountain plot heights for the 80 alignable (±)-foldback reads, 105 unalignable (±)-foldback reads, 13,762 (+)-strand reads, and 340 true (-)-strand reads. Read sequences were folded using RNAfold (Vienna RNA software suite). The mountain plot height of a structure refers to the maximum number of base-pairs enclosing any one position in a structure. Mountain plot heights were normalized for each read by dividing by the read length. The maximum normalized mountain plot height is 50%, corresponding to a perfect hairpin structure with zero unpaired bases in the loop. Larger normalized mountain plot heights correspond with structures that were more hairpin-like, and vice versa for smaller normalized mountain plot heights. Foldback and non-foldback reads exhibited inverted distributions, with foldback reads largely exhibiting hairpin-like structures.

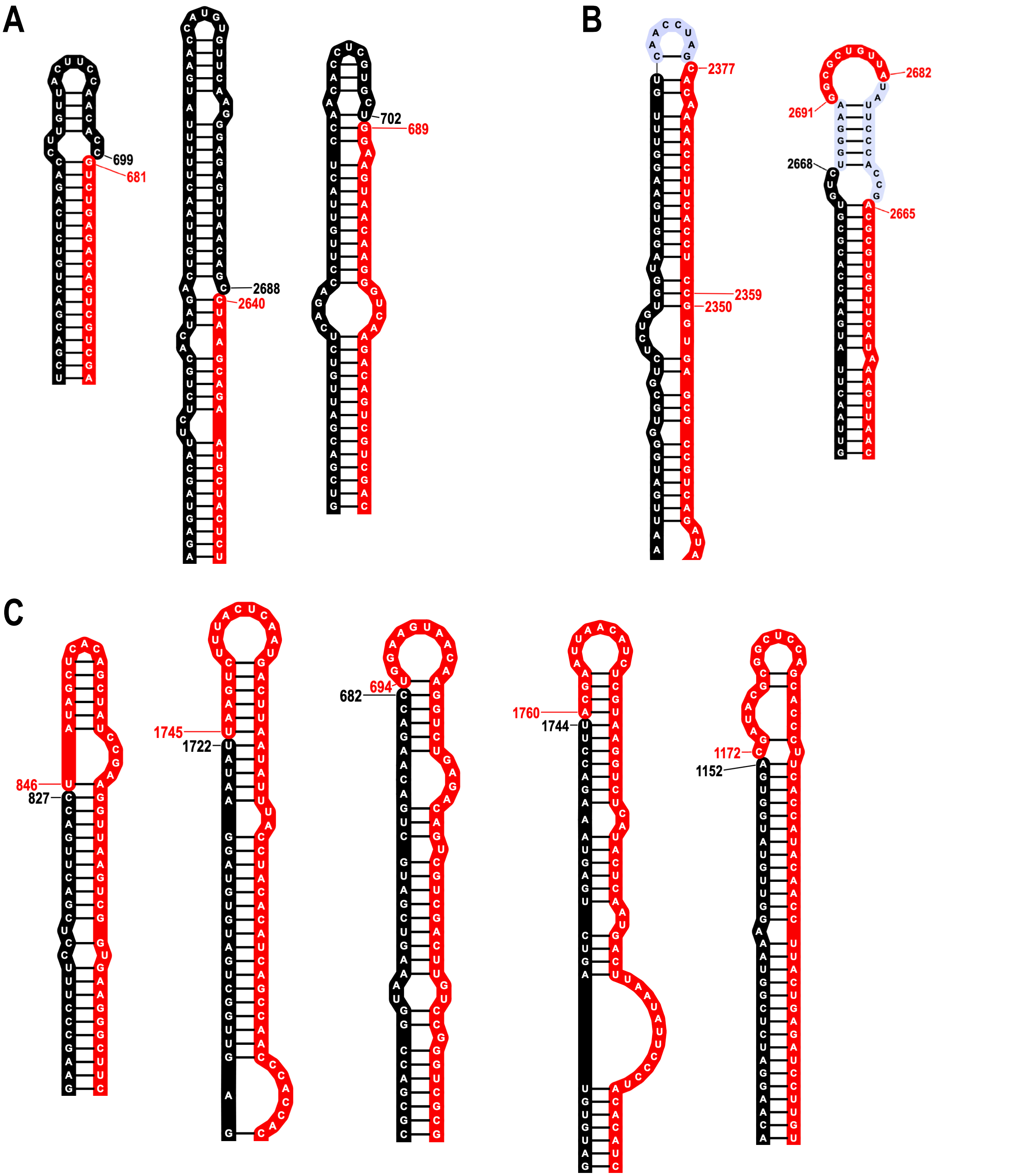

Figure S6. Examples of (+/-)-foldback loop and loop-proximal sequences. (**A**) (+/-)-foldbacks with (+)-strand sequence in the hairpin loop that extends partially down the 3´ side of the foldback stem. (+)-strand sequence in the foldbacks is shaded black, (-)-strand sequence is shaded red. (**B**) (+/-)-foldbacks possessing non-CY1 sequence between the (+)- and (-)-strands. Sequences that are apparently non-viral are shaded light blue. (**C**) (+/-)-foldbacks that contain (-)-strand sequence in their loops and partially down the 5´ sides of the foldback hairpins. Corresponding (+)-strand sequence that should be the template for these (-)-strand sequences is not present in these (+/-)-foldbacks, which disagrees with the generally held model of how (+/-)-foldbacks are generated by viral RdRp (e.g., Vignuzzi and López. 2019. *Nat Microbiol 4:1075-1087).*
